# Drug persistence and need for dose intensification to adalimumab therapy; the importance of therapeutic drug monitoring in inflammatory bowel diseases

**DOI:** 10.1186/s12876-017-0654-1

**Published:** 2017-08-08

**Authors:** Lorant Gonczi, Zsuzsanna Kurti, Mariann Rutka, Zsuzsanna Vegh, Klaudia Farkas, Barbara D. Lovasz, Petra A. Golovics, Krisztina B. Gecse, Balazs Szalay, Tamas Molnar, Peter L. Lakatos

**Affiliations:** 1 First Department of Medicine, Semmelweis University, Koranyi S 2A, Budapest, H-1083 Hungary; 2 First Department of Medicine, University of Szeged, Szeged, Hungary; 3 Institute of Applied Health Sciences, Semmelweis University, Budapest, Hungary; 4 Department of Laboratory Medicine, Semmelweis University, Budapest, Hungary; 5 Division of Gastroenterology, McGill University, MUHC, Montreal General Hospital, 1650 Ave. Cedar, D16.173. 1, Montreal, QC H3G 1A4 Canada

**Keywords:** Adalimumab, Therapeutic drug monitoring, Inflammatory bowel diseases, Loss of response

## Abstract

**Background:**

Therapeutic drug monitoring (TDM) aid therapeutic decision making in patients with inflammatory bowel disease (IBD) who lose response to anti-TNF therapy. Our aim was to evaluate the frequency and predictive factors of loss of response (LOR) to adalimumab using TDM in IBD patients.

**Methods:**

One hundred twelve IBD patients (with 214 TDM measurements, CD/UC 84/28, male/female 50/62, mean age CD/UC: 36/35 years) were enrolled in this consecutive cohort from two referral centres in Hungary. Demographic data were comprehensively collected and harmonized monitoring strategy was applied. Previous and current therapy, laboratory data and clinical activity were recorded at the time of TDM. Patients were evaluated either at the time of suspected LOR or during follow-up. TDM measurements were determined by commercial ELISA (LISA TRACKER, Theradiag, France).

**Results:**

Among 112 IBD patients, LOR/drug persistence was 25.9%/74.1%. The cumulative ADA positivity (>10 ng/mL) and low TL (<5.0 μg/mL) was 12.1% and 17.8% after 1 year and 17.3% and 29.5% after 2 years of adalimumab therapy. Dose intensification was needed in 29.5% of the patients. Female gender and ADA positivity were associated with LOR (female gender: *p* < 0.001, OR:7.8 CI 95%: 2.5–24.3, ADA positivity: *p* = 0.007 OR:3.6 CI 95%: 1.4–9.5).

**Conclusions:**

ADA development, low TL and need for dose intensification were frequent during adalimumab therapy and support the selective use of TDM in IBD patients treated with adalimumab. ADA positivity and gender were predictors of LOR.

**Electronic supplementary material:**

The online version of this article (doi:10.1186/s12876-017-0654-1) contains supplementary material, which is available to authorized users.

## Background

Inflammatory bowel diseases (IBD) are chronic conditions that significantly influence quality of life (QoL) and can lead to disability and complications. Anti-tumor necrosis factor alfa (anti-TNF) therapy is effective in IBD, demonstrating improvement in patients’ QoL, leading to clinical remission and mucosal healing, reducing the need for surgery and hospitalizations. However, approximately 10-30% of the patients do not respond to the initial treatment and app. one third of the patients lose response to anti-TNF therapy over time [1]. There are several possible causes for loss of response (LOR) to anti-TNF therapy, although one of the most common is decreased drug levels due to the development of anti-drug antibodies (ADA). ADAs can neutralize the anti-TNF drug connecting to the Fab segment of the protein or bind only the anti-TNF molecule promoting the formation of immune complexes both of them leading to increased clearance of the anti-TNF drug through the reticulo-endothelial system. This results in altered drug pharmacokinetics and in a reduction of therapeutic efficacy [2]. Therapeutic drug monitoring (TDM), measuring drug trough levels (TL) and ADA levels may aid the therapeutic decision in patients who lose response to anti-TNF therapy. Several studies have indicated that TDM based therapy may predict the loss of response to infliximab therapy [3, 4] and TDM based dosing of infliximab therapy can result in economic benefit [5].

However, less data is available on the relevance of TDM assessment during adalimumab therapy, whether TLs and ADA levels are strongly associated with disease outcome remains questionable. Although correlation between the adalimumab drug concentration and clinical outcome was reported, the role of routine TDM assessment during adalimumab therapy remained unclear [6]. In the post-hoc analysis of the CLinical Assessment of Adalimumab Safety and Efficacy Studied as Induction Therapy in Crohn’s Disease (CLASSIC) I/II trial, a positive correlation between serum adalimumab concentrations and clinical remission was reported, although it was not able to delineate a reliable therapeutic cut-off for adalimumab therapy [7]. More recent studies suggested that the optimal therapeutic TL for adalimumab is at around 5 μg/mL [8–10], while different reports exist depending on the measurement methods [2].

The aim of the present study was to evaluate the frequency and predictive factors of loss of response to adalimumab therapy and the role of TDM to predict LOR in adalimumab treated IBD patients.

## Methods

Patients were consecutively enrolled in this cohort between 2014 November and 2016 June from two referral IBD centres in Hungary. Demographic data were comprehensively collected and a harmonized monitoring strategy (including regular assessment of disease activity/biomarkers at start of the adalimumab therapy and during follow-up) was applied as requested by the Hungarian National Health Fund [11]. Previous and current therapy, laboratory data and clinical activity at the time of TDM were recorded. Adalimumab was administered at an induction dose of 160/80 mg and then at standard doses of 40 mg every other week. Dose intensification was defined as administration of 40 mg every week. Samples for TDM were collected right before routine adalimumab injection. Disease location and disease behavior in Crohn’s disease (CD) and disease extent in ulcerative colitis (UC) were classified according to the Montreal classification [12].

Patients were evaluated either at the time of suspected LOR (based on clinical evaluation) or during regular follow-up visits with TDM measurement using a conventional and bridging enzyme-linked immunosorbent (ELISA) assay (LISA TRACKER, Theradiag, France). For the measurement of ADA levels, the bridging ELISA method uses a double-antigen bridge: ADAs create a bridge between adalimumab immobilized on the plate and adalimumab enzyme-linked conjugate. ELISA measurements were performed at the Department of Laboratory Medicine, Semmelweis University, Budapest. All ELISA kit was validated for accuracy and reproducibility of TDM for adalimumab (Theradiag, France). The limit of detection for the adalimumab drug level is 0.1 μg/mL determined by the manufacturer of the assay. The optimal therapeutic cut-off value for low adalimumab TL was defined as 5 μg/ml, in concordance with previous studies. The standard cut-off value of detectable ADA levels by the assay was 10 ng/ml. To better stratify ADA positive patients we defined ADA levels more than 200 ng/ml as ‘high’ ADA level. Transient ADA was defined when the ADA positivity disappeared on the next TDM of the patient. In CD, clinical remission was defined as Crohn’s Disease Activity Index (CDAI) < 150 points or no fistula drainage as assessed by the Fistula Drainage Assessment, while clinical response was defined as a decrease in CDAI with more than 70 points or at least 50% reduction in the number and production of draining fistulas [13]. In patients with UC, the partial Mayo score (pMayo) was used: patients with more than a 3 point reduction was defined as responder, while patients with pMayo <3 were considered as being in remission [14]. LOR was defined as discontinuation of adalimumab therapy. Patients with ongoing symptoms despite dose intensification were discontinued from further adalimumab therapy, and considered to have loss of response. Patients regaining response after dose intensification were considered to have sustained clinical benefit.

### Statistical considerations

For categorical data frequency distributions were analysed, for continuous variables mean and SD were calculated. Chi-squared test was used to evaluate differences within subgroups of patients and binary logistic regression was performed for multivariate analysis (variables with a *p* < 0.2 in univariate analysis were included in the multivariate models). For time-dependent outcomes Kaplan-Meier curves were plotted was performed. A *p*-value of <0.05 was regarded as statistically significant. Statistical analysis was performed using the SPSS software v. 20.0 (Chicago, IL).

### Ethical statement

The study complies with the principles of the Declaration of Helsinki. The study protocol was approved by the Semmelweis University Regional and Institutional Committee of Science and Research Ethics. (29772-2/2014/EKU).

## Results

### Patient characteristics

One hundred twelve IBD patients (with 214 TDM measurements, CD/UC 84/28, male/female 50/62, mean age CD/UC: 36/35 years (y) (SD: 10.9/11.2 y) were enrolled in this consecutive cohort. Detailed patient characteristics are shown in Table 1.

**Table 1:** Patient characteristics

	CD	UC
Patients number (*n* = 112)	84	28
Males/females	39/45 (46%/54%)	11/17 (39%/60%)
Mean age at TDM (SD)	36 years (10.9)	35 years (11.2)
Mean duration of adalimumab therapy (SD)	157.8 weeks (101.7)	70.1 weeks (58.0)
Age at onset (<16y/17-40y/>40y)	13/64/7	-
Localisation of Crohn’s disease (ileal/colon/ileocolon/upper GI/ileocolon + upper GI)	13/16/47/2/5	-
Extension of colitis (proctitis/left sided/extensive)	-	0/19/9
Behaviour of CD (inflammatory/stricturing/penetrating/strict. + pen.)	29/18/27/10	-
Perianal disease in CD	42/84 (50%)	-
Previous surgery	43/84 (48.8%)	0/28 (0%)
Smoking (yes/no/previous)	44/24/16	19/2/7
Previous 5-ASA	69/84 (82.1%)	26/28 (93%)
Previous steroid	76/84 (90.5%)	28/28 (100%)
Previous/concomitant AZA	67/84 (80%)/42/84 (50%)	20/28 (71.4%)/2/28 (28.6%)
Previous anti-TNF	50/84 (60.2%)	23/28 (82%)
Previous IFX/ADM/both	38/5/7	22/1/0

(*CD* Crohn’s disease, *UC* Ulcerative colitis, *SD* Standard deviation, *TDM* Therapeutic drug monitoring, *GI* Gastrointestinal, *5-ASA* 5-aminosalycilate, *AZA* Azathioprine, *anti-TNF* Anti-tumor necrosis factor, *IFX* Infliximab, *ADM* Adalimumab)

The frequency of previous and current 5-aminosalicylate (5-ASA), steroid and azathioprine (AZA) exposure were 85.6%/26.8%, 92.9%/6.3% and 77.7%/44.7%. Previous anti-TNF therapy was present in 65.2% (CD 60.2%, UC 82.1%) in the IBD cohort. Mean duration of adalimumab therapy during follow-up was 157.8/70.1 weeks (SD: 101.7/58.0 weeks) in CD/UC.

### Frequency of low TL and ADA development

Among 112 IBD patients, frequency of ADA positivity was 20.5% (23/112). Among the ADA positive patients, 7 patients had high ADA titres (>200 ng/ml) and 7 patients had only transient ADA positivity (6.3%). Frequency of low TL (<5.0 μg/mL) was 31.3% (35/112). Cumulative ADA positivity was 12.1% and low TL rate was 17.8% after 1 year, and 17.3% and 29.5%, respectively after 2 years of adalimumab therapy. Concomitant immunomodulatory (IM) therapy was not associated with ADA positivity (*p* = 0.156). The combination of normal TL and no ADA, normal TL and ADA positivity, low TL and no ADA, and low TL and ADA positivity were observed in 58%, 10.7%, 21.4% and 9.8% at TDM measurement (Table 2).

**Table 2:** The combination of ADA and TL status

	Normal TL	Low TL
ADA negative	58%	21.4%
ADA positive	10.7%	9.8%

(*ADA* Anti-drug antibody, *TL* Trough level)

### Frequency of dose intensification and loss of response

**Fig. 1:**
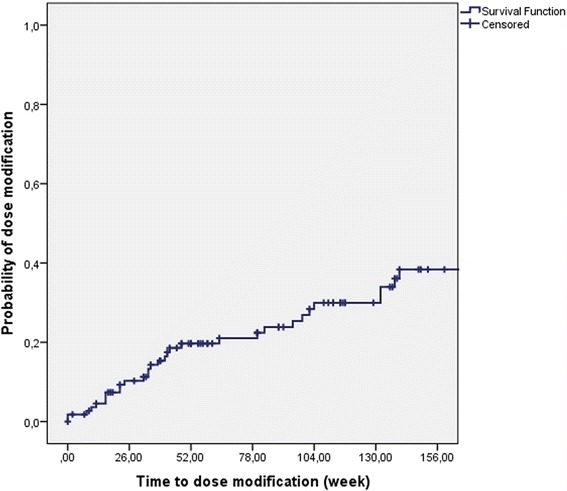
Probability of dose intensification in Kaplan-Meier analysis

**Fig. 2:**
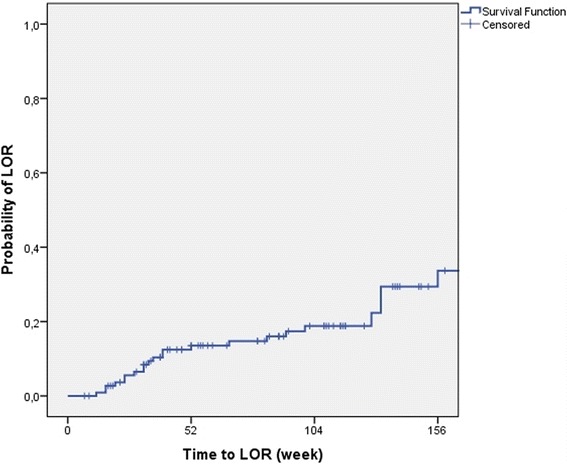
Probability of LOR in Kaplan-Meier analysis (LOR: loss of response)

Dose intensification was needed in 29.5% of patients and 25.9% of the patients had loss of response during the follow-up. The rate of LOR was similar in the two different IBD centres (25.4% and 26.5%). In Kaplan-Meier analysis, probability of dose intensification and LOR was 19.7% and 17.5% in the first year and 30% and 18.8% in the second year of adalimumab therapy (Figs. 1 and 2).

### Predictors for dose intensification, loss of response and TDM levels

Though the association between low TL and ADA positivity was statistically not significant, the trend towards an association is clear (47.8% vs. 27%, *p* = 0.054, OR: 0.4 CI 95%: 0.2–1.0), furthermore this difference was significant when analysing patients with low and high ADA titre separately. Rate of low TL in patients with high ADA was 85.7%, while it was 28.6% in patients with low ADA and 27.5% in ADA negative patients (*p* = 0.006). There was no association between TL and LOR.

ADA positivity was significantly associated with LOR (*p* = 0.007, ADA positivity in LOR 47.8% vs. 20.2%, OR: 3.6, CI 95%: 1.4 – 9.5), even if patients with low or high titre ADA (LOR rates in high ADA: 42.9%, low ADA: 50.0%, no ADA: 20.9%, *p* = 0.039, OR was not available) were analysed separately. The association was significant in CD with a similar trend in UC patients (CD: 52.9% vs. 23.9%, *p* = 0.019, OR: 3.6, CI 95%: 1.2–10.8, UC: 33.3% vs. 9%, *p* = 0.133, OR: 5.0 CI 95%: 0.5–46.7).

Transient ADA was marginally associated with LOR (*p* = 0.051, OR: 4.3, CI 95%: 0.9–20.4).

LOR was more frequent among patients with dose intensification (45.5% vs. 17.7%, *p* = 0.002, OR: 3.869, CI 95%: 1.579–9.479), especially in CD (51.9% vs. 19.3%, *p* = 0.002, OR: 4.503 CI95%: 1.655–12.258) with the same trend in UC.

There was a significant association between LOR and female gender (86.2% vs. 44.6%, *p* < 0.001, OR: 7.8 CI 95%: 2.5–24.3) both in CD and UC. In a sensitivity analysis, results remained similar if the data from the two centres were analysed separately. In contrast, there was no association between female gender and dose intensification, low TL or ADA positivity.

Gender (*p* < 0.001, OR: 9.1, 95% CI: 2.7–30.5) and ADA positivity (*p* = 0.007, OR: 4.7, 95% CI: 1.5–14.3) remained independent predictors of LOR in a multivariate analysis.

We did not find any association between previous anti-TNF therapy and LOR or ADA status.

Finally, dose intensification was associated with need of steroid therapy in patients with CD (40.7% vs. 19.6%, *p* = 0.04, OR: 2.8, CI 95%: 1.0–7.7).

## Discussion

Results from the present study suggest that ADA development, low TL and need for dose intensification are frequent during adalimumab therapy and support the use of selected TDM assessment in IBD patients during adalimumab therapy.

Need for dose intensification and frequency of LOR was 19.7% and 17.5% after 1 year and 30% and 18.8% after 2 years of adalimumab therapy. These results are in concordance with the review of Billioud et al. showing that the mean percentage of LOR and dose intensification was 18.2% and 37% with an annual risk of 20.3% and 24.8% per patient year among primary responders to adalimumab [15]. Nevertheless, in a follow-up study from Leuven, 65.4% of adalimumab treated patients with previous failure of infliximab therapy needed dose intensification and 38.5% eventually stopped adalimumab therapy mainly due to LOR [16]. In this study, LOR was more frequent in patients with low TL and high ADA levels during long-term therapy but no predictors for short term clinical response were detected [14].

In the present study, approximately one third of the patients had low TL and 20.5% had ADA positivity. Patients with high ADA titres (>200 ng/mL) had low TL in 85%. Despite of ADA development, low TL was not associated with LOR. This suggests that primarily ADA positivity should be considered as an indicator for treatment failure. Similarly, Roblin et al. suggested that low TL without ADA can be overcome by dose optimization with a high rate of clinical response (67%) after dose optimization and is not leading per se to LOR. In contrast, low TL with detectable ADA is associated with treatment failure, and switching to another anti-TNF or other therapeutic class should be considered [17]. Additional articles also suggested that the development of ADA is one of the most important mechanisms of underlying LOR, and routine TDM assessment may help improving patient outcomes. In an Italian study, low TL and ADA positivity correlated with clinical and endoscopic recurrence also in postoperative CD patients [18]. Finally, in a French study, adalimumab TL and duration of treatment were associated independently with mucosal healing, while adalimumab TL, ADA positivity, duration of treatment and adalimumab dose, but not CRP level were associated independently with clinical remission in multivariate analysis [8].

More than half of our patients received another anti-TNF agent previously (previous IFX rate: 53% in CD, 78% in UC) in the present study. We did not find any association between previous anti-TNF therapy and LOR or ADA status, however the proportion of patients with previous anti-TNF exposure was very high (74/112). In contrast, in the study of Yarur et al., detectable ADA to adalimumab was associated with low adalimumab TL, previous infliximab use, macroscopic mucosal inflammation and need for corticosteroids [19]. Similarly, the positivity rate for ADA to adalimumab was significantly higher in patients who lost response to infliximab (50%) than in those naive to anti-TNFs (12.5%) [10].

Concomitant immunomodulatory (IM) therapy was not associated with ADA positivity, in line with previous studies [9, 13, 20]. Few studies reported a benefit of parallel IMs during adalimumab therapy. In a Belgian study, time to dose escalation was longer in patients who were treated with IMs, while Yarur et al. reported that combination therapy of adalimumab and IM yielded a higher mean level of serum adalimumab compared with monotherapy (14 μg/mL vs 9 μg/mL; *p* = 0.026) [14, 17].

In the present study, there was an association between LOR and female gender both in CD and UC, even when data from the two referral centres were analysed separately. We did not find gender-related differences in other parameters (e.g. TL, ADA, and severity of disease, pervious and current therapy). In a previous retrospective study by Cohen et al. [21], gender was also associated with dose escalation during adalimumab therapy. Gender related differences were also reported in the rheumatology literature [22, 23].

Low adalimumab TL was defined as <5 μg/mL, in concordance with previous studies. However, reported cut-off values vary. Mazor et al. identified a drug TL of 5.85 μg/mL as the optimal cut-off for predicting remission (defined as asymptomatic patients with normal CRP) in CD [9]. In a Japanese study a TL of 5.9 μg/ml was identified to best predict normal CRP in a receiver operator curve (ROC) analysis [10]. Presence of ADA or a serum drug concentration lower than 5 μg/mL was associated with worse self-reported disease symptoms and elevated CRP levels [14, 17]. In contrast, Ungar et al. reported that serum levels of 8–12 μg/mL for adalimumab were required to achieve mucosal healing in patients with IBD. Adalimumab TL higher than 7.1 μg/mL predicted MH with 85% specificity in ROC analysis [20].

Current tests for anti-TNF and ADA concentrations are mostly based on enzyme immunoassays. For existing commercially available assays for antibodies, the presence of drug generally interferes with the detection of ADAs. All ADA assays are drug-sensitive to some extent because most assays use the drug itself as labeled detecting antibody. Newer assays, based on high-performance liquid chromatography or the pH shift-anti-idiotype method, which can detect ADAs in the presence of circulating drug have been developed, however most of these methods only available for infliximab and also not accessible in everyday clinical practice [24, 25].

The strengths of the present study are the harmonized monitoring strategy, prospective collection of clinical and laboratory data. Limitations of the study include the relatively small cohort size, especially in UC. Multiple TDM samples were available only in few patients. Of note, unlike infliximab, there are very few studies evaluating serial TDM measurements in adalimumab therapy. Further investigation is needed applying serial/routine TDM measurements to determine the exact role and usability of routine TDM in adalimumab therapy. In addition, whether TDM for adalimumab needs to be performed at trough remains conflictive in current literature and definitely requires more research. Of note, in a very recent paper by Ward et al. author have shown only little variation in drug levels during maintenance adalimumab therapy assessed on days 4-6, 7-9 and 13-14 (trough level). Importantly, drug levels on day 9 were the best predictor of a therapeutic drug trough level [26].

## Conclusions

The present study suggests that ADA development, low TL and need for dose intensification are frequent during adalimumab therapy and our results support the use of selective TDM assessment in IBD patients on adalimumab therapy. ADA positivity was identified as predictor of loss of response.

## Additional file

Additional file 1:
*Data set.* We provided the dataset of our research as requested by the guidelines of BMC Gastroenterology. All available result of our dataset is presented in the manuscript. (DOCX 90 kb)

## Abbreviations

ADA: Anti-drug antibody
anti-TNF: Anti-tumor necrosis factor alfa
CD: Crohn’s disease
CDAI: Crohn’s Disease Activity Index
ELISA: Enzyme-linked immunosorbent assay
IBD: Inflammatory bowel disease
LOR: Loss of response
pMayo: Partial Mayo Score
QoL: Quality of life
TDM: Therapeutic drug monitoring
TL: Trough level
UC: Ulcerative colitis
