# Supplementary material for: Drug persistence and need for dose intensification to adalimumab therapy; the importance of therapeutic drug monitoring in inflammatory bowel diseases
Source: BMC Gastroenterol. 2017 Aug 8;17:97. doi: 10.1186/s12876-017-0654-1 (PMC5549364; doi:10.1186/s12876-017-0654-1)
Supplement: Additional file 1: — Data set. We provided the dataset of our research as requested by the guidelines of BMC Gastroenterology. All available result of our dataset is presented in the manuscript. (DOCX 90 kb) [file 12876_2017_654_MOESM1_ESM.docx]

**Kaplan-Meier**

| **Case Processing Summary** | | | |
| --- | --- | --- | --- |
| Total N | N of Events | Censored | |
|  |  | N | Percent |
| 112 | 29 | 83 | 74,1% |

| **Survival Table** | | | | | | |
| --- | --- | --- | --- | --- | --- | --- |
|  | Time | Status | Cumulative Proportion Surviving at the Time | | N of Cumulative Events | N of Remaining Cases |
|  |  |  | Estimate | Std. Error |  |  |
| 1 | 7,000 | ,00 | . | . | 0 | 111 |
| 2 | 9,000 | ,00 | . | . | 0 | 110 |
| 3 | 12,000 | 1,00 | ,991 | ,009 | 1 | 109 |
| 4 | 16,000 | 1,00 | . | . | 2 | 108 |
| 5 | 16,000 | 1,00 | ,973 | ,016 | 3 | 107 |
| 6 | 17,000 | ,00 | . | . | 3 | 106 |
| 7 | 18,000 | ,00 | . | . | 3 | 105 |
| 8 | 19,000 | ,00 | . | . | 3 | 104 |
| 9 | 20,000 | 1,00 | ,963 | ,018 | 4 | 103 |
| 10 | 22,000 | ,00 | . | . | 4 | 102 |
| 11 | 24,000 | 1,00 | . | . | 5 | 101 |
| 12 | 24,000 | 1,00 | ,944 | ,022 | 6 | 100 |
| 13 | 28,000 | 1,00 | ,935 | ,024 | 7 | 99 |
| 14 | 29,000 | ,00 | . | . | 7 | 98 |
| 15 | 32,000 | 1,00 | . | . | 8 | 97 |
| 16 | 32,000 | 1,00 | ,916 | ,027 | 9 | 96 |
| 17 | 32,000 | ,00 | . | . | 9 | 95 |
| 18 | 32,000 | ,00 | . | . | 9 | 94 |
| 19 | 33,000 | ,00 | . | . | 9 | 93 |
| 20 | 34,000 | 1,00 | ,906 | ,028 | 10 | 92 |
| 21 | 35,000 | ,00 | . | . | 10 | 91 |
| 22 | 36,000 | 1,00 | ,896 | ,030 | 11 | 90 |
| 23 | 39,000 | ,00 | . | . | 11 | 89 |
| 24 | 39,000 | ,00 | . | . | 11 | 88 |
| 25 | 39,000 | ,00 | . | . | 11 | 87 |
| 26 | 40,000 | 1,00 | . | . | 12 | 86 |
| 27 | 40,000 | 1,00 | ,876 | ,032 | 13 | 85 |
| 28 | 42,000 | ,00 | . | . | 13 | 84 |
| 29 | 43,000 | ,00 | . | . | 13 | 83 |
| 30 | 46,000 | ,00 | . | . | 13 | 82 |
| 31 | 48,000 | ,00 | . | . | 13 | 81 |
| 32 | 48,000 | ,00 | . | . | 13 | 80 |
| 33 | 52,000 | 1,00 | ,865 | ,034 | 14 | 79 |
| 34 | 52,000 | ,00 | . | . | 14 | 78 |
| 35 | 52,000 | ,00 | . | . | 14 | 77 |
| 36 | 55,000 | ,00 | . | . | 14 | 76 |
| 37 | 56,000 | ,00 | . | . | 14 | 75 |
| 38 | 57,000 | ,00 | . | . | 14 | 74 |
| 39 | 59,000 | ,00 | . | . | 14 | 73 |
| 40 | 61,000 | ,00 | . | . | 14 | 72 |
| 41 | 67,000 | ,00 | . | . | 14 | 71 |
| 42 | 68,000 | 1,00 | ,852 | ,035 | 15 | 70 |
| 43 | 80,000 | ,00 | . | . | 15 | 69 |
| 44 | 80,000 | ,00 | . | . | 15 | 68 |
| 45 | 83,000 | ,00 | . | . | 15 | 67 |
| 46 | 84,000 | 1,00 | ,840 | ,037 | 16 | 66 |
| 47 | 85,000 | ,00 | . | . | 16 | 65 |
| 48 | 89,000 | ,00 | . | . | 16 | 64 |
| 49 | 89,000 | ,00 | . | . | 16 | 63 |
| 50 | 89,000 | ,00 | . | . | 16 | 62 |
| 51 | 91,000 | ,00 | . | . | 16 | 61 |
| 52 | 92,000 | 1,00 | ,826 | ,039 | 17 | 60 |
| 53 | 93,000 | ,00 | . | . | 17 | 59 |
| 54 | 100,000 | 1,00 | ,812 | ,041 | 18 | 58 |
| 55 | 102,000 | ,00 | . | . | 18 | 57 |
| 56 | 108,000 | ,00 | . | . | 18 | 56 |
| 57 | 108,000 | ,00 | . | . | 18 | 55 |
| 58 | 109,000 | ,00 | . | . | 18 | 54 |
| 59 | 110,000 | ,00 | . | . | 18 | 53 |
| 60 | 112,000 | ,00 | . | . | 18 | 52 |
| 61 | 115,000 | ,00 | . | . | 18 | 51 |
| 62 | 115,000 | ,00 | . | . | 18 | 50 |
| 63 | 116,000 | ,00 | . | . | 18 | 49 |
| 64 | 117,000 | ,00 | . | . | 18 | 48 |
| 65 | 117,000 | ,00 | . | . | 18 | 47 |
| 66 | 125,000 | ,00 | . | . | 18 | 46 |
| 67 | 128,000 | 1,00 | . | . | 19 | 45 |
| 68 | 128,000 | 1,00 | ,777 | ,046 | 20 | 44 |
| 69 | 132,000 | 1,00 | . | . | 21 | 43 |
| 70 | 132,000 | 1,00 | . | . | 22 | 42 |
| 71 | 132,000 | 1,00 | . | . | 23 | 41 |
| 72 | 132,000 | 1,00 | ,706 | ,054 | 24 | 40 |
| 73 | 137,000 | ,00 | . | . | 24 | 39 |
| 74 | 138,000 | ,00 | . | . | 24 | 38 |
| 75 | 139,000 | ,00 | . | . | 24 | 37 |
| 76 | 140,000 | ,00 | . | . | 24 | 36 |
| 77 | 148,000 | ,00 | . | . | 24 | 35 |
| 78 | 149,000 | ,00 | . | . | 24 | 34 |
| 79 | 152,000 | ,00 | . | . | 24 | 33 |
| 80 | 156,000 | 1,00 | . | . | 25 | 32 |
| 81 | 156,000 | 1,00 | ,663 | ,058 | 26 | 31 |
| 82 | 159,000 | ,00 | . | . | 26 | 30 |
| 83 | 166,000 | ,00 | . | . | 26 | 29 |
| 84 | 169,000 | ,00 | . | . | 26 | 28 |
| 85 | 170,000 | ,00 | . | . | 26 | 27 |
| 86 | 177,000 | ,00 | . | . | 26 | 26 |
| 87 | 180,000 | ,00 | . | . | 26 | 25 |
| 88 | 180,000 | ,00 | . | . | 26 | 24 |
| 89 | 185,000 | ,00 | . | . | 26 | 23 |
| 90 | 187,000 | ,00 | . | . | 26 | 22 |
| 91 | 190,000 | 1,00 | ,633 | ,063 | 27 | 21 |
| 92 | 196,000 | ,00 | . | . | 27 | 20 |
| 93 | 197,000 | ,00 | . | . | 27 | 19 |
| 94 | 197,000 | ,00 | . | . | 27 | 18 |
| 95 | 224,000 | ,00 | . | . | 27 | 17 |
| 96 | 239,000 | ,00 | . | . | 27 | 16 |
| 97 | 243,000 | ,00 | . | . | 27 | 15 |
| 98 | 247,000 | ,00 | . | . | 27 | 14 |
| 99 | 264,000 | 1,00 | ,588 | ,073 | 28 | 13 |
| 100 | 280,000 | 1,00 | ,543 | ,080 | 29 | 12 |
| 101 | 287,000 | ,00 | . | . | 29 | 11 |
| 102 | 295,000 | ,00 | . | . | 29 | 10 |
| 103 | 296,000 | ,00 | . | . | 29 | 9 |
| 104 | 299,000 | ,00 | . | . | 29 | 8 |
| 105 | 334,000 | ,00 | . | . | 29 | 7 |
| 106 | 336,000 | ,00 | . | . | 29 | 6 |
| 107 | 343,000 | ,00 | . | . | 29 | 5 |
| 108 | 378,000 | ,00 | . | . | 29 | 4 |
| 109 | 383,000 | ,00 | . | . | 29 | 3 |
| 110 | 386,000 | ,00 | . | . | 29 | 2 |
| 111 | 408,000 | ,00 | . | . | 29 | 1 |
| 112 | 438,000 | ,00 | . | . | 29 | 0 |

| **Means and Medians for Survival Time** | | | | | |
| --- | --- | --- | --- | --- | --- |
| Mean^a^ | | | | Median | |
| Estimate | Std. Error | 95% Confidence Interval | | Estimate | Std. Error |
|  |  | Lower Bound | Upper Bound |  |  |
| 297,090 | 21,401 | 255,144 | 339,036 | . | . |

| **Means and Medians for Survival Time** | |
| --- | --- |
| Median^a^ | |
| 95% Confidence Interval | |
| Lower Bound | Upper Bound |
| . | . |

| a. Estimation is limited to the largest survival time if it is censored. |
| --- |


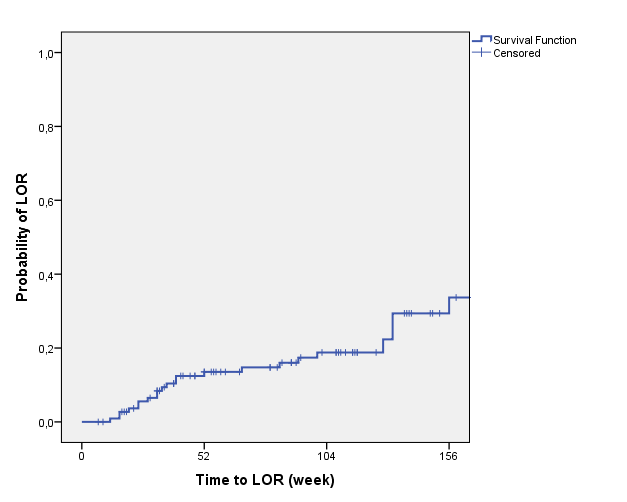


**Kaplan-Meier**

| **Case Processing Summary** | | | |
| --- | --- | --- | --- |
| Total N | N of Events | Censored | |
|  |  | N | Percent |
| 112 | 33 | 79 | 70,5% |

| **Survival Table** | | | | | | |
| --- | --- | --- | --- | --- | --- | --- |
|  | Time | Status | Cumulative Proportion Surviving at the Time | | N of Cumulative Events | N of Remaining Cases |
|  |  |  | Estimate | Std. Error |  |  |
| 1 | ,000 | 1,00 | . | . | 1 | 111 |
| 2 | ,000 | 1,00 | ,982 | ,013 | 2 | 110 |
| 3 | ,000 | ,00 | . | . | 2 | 109 |
| 4 | 2,000 | ,00 | . | . | 2 | 108 |
| 5 | 7,000 | ,00 | . | . | 2 | 107 |
| 6 | 8,000 | 1,00 | ,973 | ,015 | 3 | 106 |
| 7 | 9,000 | ,00 | . | . | 3 | 105 |
| 8 | 10,000 | 1,00 | ,964 | ,018 | 4 | 104 |
| 9 | 12,000 | 1,00 | ,954 | ,020 | 5 | 103 |
| 10 | 12,000 | ,00 | . | . | 5 | 102 |
| 11 | 16,000 | 1,00 | . | . | 6 | 101 |
| 12 | 16,000 | 1,00 | . | . | 7 | 100 |
| 13 | 16,000 | 1,00 | ,926 | ,025 | 8 | 99 |
| 14 | 17,000 | ,00 | . | . | 8 | 98 |
| 15 | 18,000 | ,00 | . | . | 8 | 97 |
| 16 | 19,000 | ,00 | . | . | 8 | 96 |
| 17 | 22,000 | 1,00 | . | . | 9 | 95 |
| 18 | 22,000 | 1,00 | ,907 | ,028 | 10 | 94 |
| 19 | 22,000 | ,00 | . | . | 10 | 93 |
| 20 | 24,000 | 1,00 | ,897 | ,029 | 11 | 92 |
| 21 | 28,000 | ,00 | . | . | 11 | 91 |
| 22 | 32,000 | 1,00 | ,887 | ,031 | 12 | 90 |
| 23 | 32,000 | ,00 | . | . | 12 | 89 |
| 24 | 32,000 | ,00 | . | . | 12 | 88 |
| 25 | 33,000 | ,00 | . | . | 12 | 87 |
| 26 | 34,000 | 1,00 | . | . | 13 | 86 |
| 27 | 34,000 | 1,00 | ,867 | ,033 | 14 | 85 |
| 28 | 35,000 | 1,00 | ,857 | ,034 | 15 | 84 |
| 29 | 35,000 | ,00 | . | . | 15 | 83 |
| 30 | 38,000 | 1,00 | ,847 | ,035 | 16 | 82 |
| 31 | 39,000 | ,00 | . | . | 16 | 81 |
| 32 | 39,000 | ,00 | . | . | 16 | 80 |
| 33 | 39,000 | ,00 | . | . | 16 | 79 |
| 34 | 41,000 | 1,00 | ,836 | ,037 | 17 | 78 |
| 35 | 42,000 | 1,00 | ,825 | ,038 | 18 | 77 |
| 36 | 42,000 | ,00 | . | . | 18 | 76 |
| 37 | 43,000 | 1,00 | ,814 | ,039 | 19 | 75 |
| 38 | 43,000 | ,00 | . | . | 19 | 74 |
| 39 | 46,000 | ,00 | . | . | 19 | 73 |
| 40 | 48,000 | 1,00 | ,803 | ,040 | 20 | 72 |
| 41 | 48,000 | ,00 | . | . | 20 | 71 |
| 42 | 48,000 | ,00 | . | . | 20 | 70 |
| 43 | 51,000 | ,00 | . | . | 20 | 69 |
| 44 | 52,000 | ,00 | . | . | 20 | 68 |
| 45 | 52,000 | ,00 | . | . | 20 | 67 |
| 46 | 52,000 | ,00 | . | . | 20 | 66 |
| 47 | 55,000 | ,00 | . | . | 20 | 65 |
| 48 | 56,000 | ,00 | . | . | 20 | 64 |
| 49 | 57,000 | ,00 | . | . | 20 | 63 |
| 50 | 59,000 | ,00 | . | . | 20 | 62 |
| 51 | 59,000 | ,00 | . | . | 20 | 61 |
| 52 | 61,000 | ,00 | . | . | 20 | 60 |
| 53 | 64,000 | 1,00 | ,790 | ,041 | 21 | 59 |
| 54 | 64,000 | ,00 | . | . | 21 | 58 |
| 55 | 80,000 | 1,00 | ,776 | ,043 | 22 | 57 |
| 56 | 80,000 | ,00 | . | . | 22 | 56 |
| 57 | 80,000 | ,00 | . | . | 22 | 55 |
| 58 | 80,000 | ,00 | . | . | 22 | 54 |
| 59 | 83,000 | 1,00 | ,762 | ,044 | 23 | 53 |
| 60 | 89,000 | ,00 | . | . | 23 | 52 |
| 61 | 89,000 | ,00 | . | . | 23 | 51 |
| 62 | 91,000 | ,00 | . | . | 23 | 50 |
| 63 | 95,000 | 1,00 | ,746 | ,046 | 24 | 49 |
| 64 | 99,000 | 1,00 | ,731 | ,047 | 25 | 48 |
| 65 | 102,000 | 1,00 | ,716 | ,049 | 26 | 47 |
| 66 | 102,000 | ,00 | . | . | 26 | 46 |
| 67 | 104,000 | 1,00 | ,700 | ,050 | 27 | 45 |
| 68 | 108,000 | ,00 | . | . | 27 | 44 |
| 69 | 110,000 | ,00 | . | . | 27 | 43 |
| 70 | 112,000 | ,00 | . | . | 27 | 42 |
| 71 | 112,000 | ,00 | . | . | 27 | 41 |
| 72 | 115,000 | ,00 | . | . | 27 | 40 |
| 73 | 115,000 | ,00 | . | . | 27 | 39 |
| 74 | 116,000 | ,00 | . | . | 27 | 38 |
| 75 | 117,000 | ,00 | . | . | 27 | 37 |
| 76 | 117,000 | ,00 | . | . | 27 | 36 |
| 77 | 129,000 | ,00 | . | . | 27 | 35 |
| 78 | 132,000 | 1,00 | . | . | 28 | 34 |
| 79 | 132,000 | 1,00 | ,660 | ,055 | 29 | 33 |
| 80 | 136,000 | ,00 | . | . | 29 | 32 |
| 81 | 137,000 | ,00 | . | . | 29 | 31 |
| 82 | 138,000 | 1,00 | ,639 | ,057 | 30 | 30 |
| 83 | 138,000 | ,00 | . | . | 30 | 29 |
| 84 | 139,000 | ,00 | . | . | 30 | 28 |
| 85 | 140,000 | 1,00 | ,616 | ,059 | 31 | 27 |
| 86 | 140,000 | ,00 | . | . | 31 | 26 |
| 87 | 148,000 | ,00 | . | . | 31 | 25 |
| 88 | 149,000 | ,00 | . | . | 31 | 24 |
| 89 | 152,000 | ,00 | . | . | 31 | 23 |
| 90 | 159,000 | ,00 | . | . | 31 | 22 |
| 91 | 166,000 | ,00 | . | . | 31 | 21 |
| 92 | 170,000 | ,00 | . | . | 31 | 20 |
| 93 | 196,000 | ,00 | . | . | 31 | 19 |
| 94 | 197,000 | ,00 | . | . | 31 | 18 |
| 95 | 197,000 | ,00 | . | . | 31 | 17 |
| 96 | 197,000 | ,00 | . | . | 31 | 16 |
| 97 | 238,000 | ,00 | . | . | 31 | 15 |
| 98 | 238,000 | ,00 | . | . | 31 | 14 |
| 99 | 239,000 | ,00 | . | . | 31 | 13 |
| 100 | 243,000 | ,00 | . | . | 31 | 12 |
| 101 | 247,000 | ,00 | . | . | 31 | 11 |
| 102 | 254,000 | 1,00 | ,560 | ,076 | 32 | 10 |
| 103 | 280,000 | 1,00 | ,504 | ,087 | 33 | 9 |
| 104 | 280,000 | ,00 | . | . | 33 | 8 |
| 105 | 334,000 | ,00 | . | . | 33 | 7 |
| 106 | 336,000 | ,00 | . | . | 33 | 6 |
| 107 | 343,000 | ,00 | . | . | 33 | 5 |
| 108 | 378,000 | ,00 | . | . | 33 | 4 |
| 109 | 383,000 | ,00 | . | . | 33 | 3 |
| 110 | 386,000 | ,00 | . | . | 33 | 2 |
| 111 | 408,000 | ,00 | . | . | 33 | 1 |
| 112 | 438,000 | ,00 | . | . | 33 | 0 |

| **Means and Medians for Survival Time** | | | | | |
| --- | --- | --- | --- | --- | --- |
| Mean^a^ | | | | Median | |
| Estimate | Std. Error | 95% Confidence Interval | | Estimate | Std. Error |
|  |  | Lower Bound | Upper Bound |  |  |
| 276,493 | 22,938 | 231,535 | 321,452 | . | . |

| **Means and Medians for Survival Time** | |
| --- | --- |
| Median^a^ | |
| 95% Confidence Interval | |
| Lower Bound | Upper Bound |
| . | . |

| a. Estimation is limited to the largest survival time if it is censored. |
| --- |


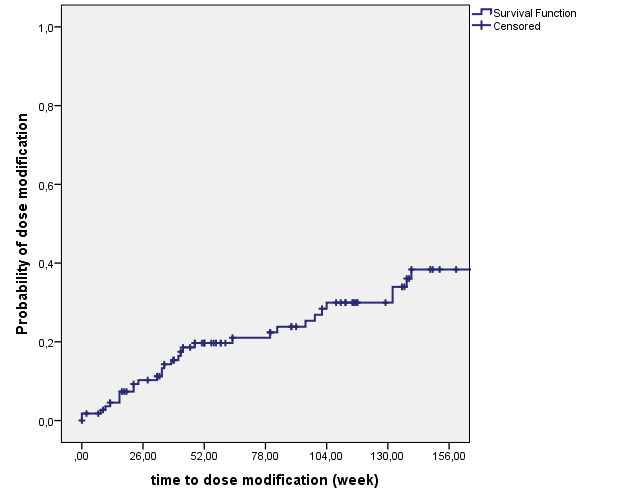


KM TLminusido_kovetes_het

/STATUS=TLminus(1)

/PRINT TABLE MEAN

/PLOT OMS.

| **ASA** | | | | | |
| --- | --- | --- | --- | --- | --- |
|  | | Frequency | Percent | Valid Percent | Cumulative Percent |
| Valid | ,00 | 16 | 14,3 | 14,4 | 14,4 |
|  | 1,00 | 95 | 84,8 | 85,6 | 100,0 |
|  | Total | 111 | 99,1 | 100,0 |  |
| Missing | System | 1 | ,9 |  |  |
| Total | | 112 | 100,0 |  |  |

| **szter** | | | | | |
| --- | --- | --- | --- | --- | --- |
|  | | Frequency | Percent | Valid Percent | Cumulative Percent |
| Valid | ,00 | 8 | 7,1 | 7,1 | 7,1 |
|  | 1,00 | 104 | 92,9 | 92,9 | 100,0 |
|  | Total | 112 | 100,0 | 100,0 |  |

| **AZA** | | | | | |
| --- | --- | --- | --- | --- | --- |
|  | | Frequency | Percent | Valid Percent | Cumulative Percent |
| Valid | ,00 | 25 | 22,3 | 22,3 | 22,3 |
|  | 1,00 | 87 | 77,7 | 77,7 | 100,0 |
|  | Total | 112 | 100,0 | 100,0 |  |

| **korabbaTNF** | | | | | |
| --- | --- | --- | --- | --- | --- |
|  | | Frequency | Percent | Valid Percent | Cumulative Percent |
| Valid | ,00 | 33 | 39,3 | 39,8 | 39,8 |
|  | 1,00 | 50 | 59,5 | 60,2 | 100,0 |
|  | Total | 83 | 98,8 | 100,0 |  |
| Missing | System | 1 | 1,2 |  |  |
| Total | | 84 | 100,0 |  |  |
| **korabbaTNF** | | | | | |
|  | | Frequency | Percent | Valid Percent | Cumulative Percent |
| Valid | ,00 | 5 | 17,9 | 17,9 | 17,9 |
|  | 1,00 | 23 | 82,1 | 82,1 | 100,0 |
|  | Total | 28 | 100,0 | 100,0 |  |
| **ADApozzz** | | | | | |
|  | | Frequency | Percent | Valid Percent | Cumulative Percent |
| Valid | ,00 | 89 | 79,5 | 79,5 | 79,5 |
|  | 1,00 | 23 | 20,5 | 20,5 | 100,0 |
|  | Total | 112 | 100,0 | 100,0 |  |
| **TLminus** | | | | | |
|  | | Frequency | Percent | Valid Percent | Cumulative Percent |
| Valid | ,00 | 35 | 31,3 | 31,3 | 31,3 |
|  | 1,00 | 77 | 68,8 | 68,8 | 100,0 |
|  | Total | 112 | 100,0 | 100,0 |  |

| **Case Processing Summary** | | | |
| --- | --- | --- | --- |
| Total N | N of Events | Censored | |
|  |  | N | Percent |
| 112 | 23 | 89 | 79,5% |

| **Survival Table** | | | | | | |
| --- | --- | --- | --- | --- | --- | --- |
|  | Time | Status | Cumulative Proportion Surviving at the Time | | N of Cumulative Events | N of Remaining Cases |
|  |  |  | Estimate | Std. Error |  |  |
| 1 | ,000 | 1,00 | . | . | 1 | 111 |
| 2 | ,000 | 1,00 | ,982 | ,013 | 2 | 110 |
| 3 | 2,000 | 1,00 | . | . | 3 | 109 |
| 4 | 2,000 | 1,00 | ,964 | ,018 | 4 | 108 |
| 5 | 2,000 | ,00 | . | . | 4 | 107 |
| 6 | 3,000 | 1,00 | ,955 | ,020 | 5 | 106 |
| 7 | 7,000 | ,00 | . | . | 5 | 105 |
| 8 | 8,000 | 1,00 | . | . | 6 | 104 |
| 9 | 8,000 | 1,00 | ,937 | ,023 | 7 | 103 |
| 10 | 9,000 | ,00 | . | . | 7 | 102 |
| 11 | 10,000 | 1,00 | ,928 | ,025 | 8 | 101 |
| 12 | 12,000 | ,00 | . | . | 8 | 100 |
| 13 | 17,000 | ,00 | . | . | 8 | 99 |
| 14 | 19,000 | ,00 | . | . | 8 | 98 |
| 15 | 21,000 | 1,00 | ,918 | ,026 | 9 | 97 |
| 16 | 22,000 | ,00 | . | . | 9 | 96 |
| 17 | 24,000 | 1,00 | ,909 | ,028 | 10 | 95 |
| 18 | 32,000 | 1,00 | ,899 | ,029 | 11 | 94 |
| 19 | 32,000 | ,00 | . | . | 11 | 93 |
| 20 | 35,000 | ,00 | . | . | 11 | 92 |
| 21 | 39,000 | ,00 | . | . | 11 | 91 |
| 22 | 39,000 | ,00 | . | . | 11 | 90 |
| 23 | 39,000 | ,00 | . | . | 11 | 89 |
| 24 | 40,000 | 1,00 | ,889 | ,030 | 12 | 88 |
| 25 | 42,000 | ,00 | . | . | 12 | 87 |
| 26 | 43,000 | ,00 | . | . | 12 | 86 |
| 27 | 44,000 | 1,00 | ,879 | ,032 | 13 | 85 |
| 28 | 48,000 | ,00 | . | . | 13 | 84 |
| 29 | 48,000 | ,00 | . | . | 13 | 83 |
| 30 | 51,000 | ,00 | . | . | 13 | 82 |
| 31 | 52,000 | ,00 | . | . | 13 | 81 |
| 32 | 52,000 | ,00 | . | . | 13 | 80 |
| 33 | 55,000 | ,00 | . | . | 13 | 79 |
| 34 | 56,000 | 1,00 | ,868 | ,033 | 14 | 78 |
| 35 | 56,000 | ,00 | . | . | 14 | 77 |
| 36 | 57,000 | ,00 | . | . | 14 | 76 |
| 37 | 59,000 | ,00 | . | . | 14 | 75 |
| 38 | 59,000 | ,00 | . | . | 14 | 74 |
| 39 | 61,000 | ,00 | . | . | 14 | 73 |
| 40 | 64,000 | ,00 | . | . | 14 | 72 |
| 41 | 67,000 | ,00 | . | . | 14 | 71 |
| 42 | 76,000 | ,00 | . | . | 14 | 70 |
| 43 | 80,000 | ,00 | . | . | 14 | 69 |
| 44 | 80,000 | ,00 | . | . | 14 | 68 |
| 45 | 80,000 | ,00 | . | . | 14 | 67 |
| 46 | 83,000 | ,00 | . | . | 14 | 66 |
| 47 | 89,000 | ,00 | . | . | 14 | 65 |
| 48 | 90,000 | 1,00 | ,854 | ,035 | 15 | 64 |
| 49 | 91,000 | ,00 | . | . | 15 | 63 |
| 50 | 91,000 | ,00 | . | . | 15 | 62 |
| 51 | 92,000 | 1,00 | . | . | 16 | 61 |
| 52 | 92,000 | 1,00 | ,827 | ,039 | 17 | 60 |
| 53 | 93,000 | ,00 | . | . | 17 | 59 |
| 54 | 99,000 | ,00 | . | . | 17 | 58 |
| 55 | 100,000 | ,00 | . | . | 17 | 57 |
| 56 | 102,000 | ,00 | . | . | 17 | 56 |
| 57 | 105,000 | ,00 | . | . | 17 | 55 |
| 58 | 108,000 | ,00 | . | . | 17 | 54 |
| 59 | 108,000 | ,00 | . | . | 17 | 53 |
| 60 | 110,000 | 1,00 | ,811 | ,041 | 18 | 52 |
| 61 | 110,000 | ,00 | . | . | 18 | 51 |
| 62 | 112,000 | 1,00 | ,795 | ,043 | 19 | 50 |
| 63 | 112,000 | ,00 | . | . | 19 | 49 |
| 64 | 115,000 | ,00 | . | . | 19 | 48 |
| 65 | 116,000 | ,00 | . | . | 19 | 47 |
| 66 | 117,000 | ,00 | . | . | 19 | 46 |
| 67 | 117,000 | ,00 | . | . | 19 | 45 |
| 68 | 125,000 | ,00 | . | . | 19 | 44 |
| 69 | 132,000 | 1,00 | ,777 | ,046 | 20 | 43 |
| 70 | 137,000 | ,00 | . | . | 20 | 42 |
| 71 | 138,000 | ,00 | . | . | 20 | 41 |
| 72 | 139,000 | ,00 | . | . | 20 | 40 |
| 73 | 140,000 | ,00 | . | . | 20 | 39 |
| 74 | 145,000 | ,00 | . | . | 20 | 38 |
| 75 | 148,000 | ,00 | . | . | 20 | 37 |
| 76 | 149,000 | ,00 | . | . | 20 | 36 |
| 77 | 152,000 | ,00 | . | . | 20 | 35 |
| 78 | 159,000 | ,00 | . | . | 20 | 34 |
| 79 | 166,000 | ,00 | . | . | 20 | 33 |
| 80 | 169,000 | ,00 | . | . | 20 | 32 |
| 81 | 169,000 | ,00 | . | . | 20 | 31 |
| 82 | 170,000 | ,00 | . | . | 20 | 30 |
| 83 | 179,000 | ,00 | . | . | 20 | 29 |
| 84 | 180,000 | ,00 | . | . | 20 | 28 |
| 85 | 180,000 | ,00 | . | . | 20 | 27 |
| 86 | 185,000 | ,00 | . | . | 20 | 26 |
| 87 | 187,000 | ,00 | . | . | 20 | 25 |
| 88 | 196,000 | ,00 | . | . | 20 | 24 |
| 89 | 196,000 | ,00 | . | . | 20 | 23 |
| 90 | 197,000 | ,00 | . | . | 20 | 22 |
| 91 | 197,000 | ,00 | . | . | 20 | 21 |
| 92 | 197,000 | ,00 | . | . | 20 | 20 |
| 93 | 202,000 | 1,00 | ,738 | ,058 | 21 | 19 |
| 94 | 216,000 | 1,00 | ,700 | ,067 | 22 | 18 |
| 95 | 224,000 | ,00 | . | . | 22 | 17 |
| 96 | 238,000 | ,00 | . | . | 22 | 16 |
| 97 | 239,000 | ,00 | . | . | 22 | 15 |
| 98 | 239,000 | ,00 | . | . | 22 | 14 |
| 99 | 243,000 | ,00 | . | . | 22 | 13 |
| 100 | 247,000 | ,00 | . | . | 22 | 12 |
| 101 | 280,000 | ,00 | . | . | 22 | 11 |
| 102 | 287,000 | ,00 | . | . | 22 | 10 |
| 103 | 295,000 | ,00 | . | . | 22 | 9 |
| 104 | 299,000 | ,00 | . | . | 22 | 8 |
| 105 | 334,000 | ,00 | . | . | 22 | 7 |
| 106 | 336,000 | ,00 | . | . | 22 | 6 |
| 107 | 343,000 | ,00 | . | . | 22 | 5 |
| 108 | 370,000 | 1,00 | ,560 | ,136 | 23 | 4 |
| 109 | 378,000 | ,00 | . | . | 23 | 3 |
| 110 | 386,000 | ,00 | . | . | 23 | 2 |
| 111 | 408,000 | ,00 | . | . | 23 | 1 |
| 112 | 438,000 | ,00 | . | . | 23 | 0 |

| **Means and Medians for Survival Time** | | | | | |
| --- | --- | --- | --- | --- | --- |
| Mean^a^ | | | | Median | |
| Estimate | Std. Error | 95% Confidence Interval | | Estimate | Std. Error |
|  |  | Lower Bound | Upper Bound |  |  |
| 325,252 | 21,047 | 284,000 | 366,504 | . | . |

| **Means and Medians for Survival Time** | |
| --- | --- |
| Median^a^ | |
| 95% Confidence Interval | |
| Lower Bound | Upper Bound |
| . | . |

| a. Estimation is limited to the largest survival time if it is censored. |
| --- |

| **Case Processing Summary** | | | |
| --- | --- | --- | --- |
| Total N | N of Events | Censored | |
|  |  | N | Percent |
| 112 | 77 | 35 | 31,3% |

| **Survival Table** | | | | | | |
| --- | --- | --- | --- | --- | --- | --- |
|  | Time | Status | Cumulative Proportion Surviving at the Time | | N of Cumulative Events | N of Remaining Cases |
|  |  |  | Estimate | Std. Error |  |  |
| 1 | 2,000 | 1,00 | ,991 | ,009 | 1 | 111 |
| 2 | 2,000 | ,00 | . | . | 1 | 110 |
| 3 | 4,000 | ,00 | . | . | 1 | 109 |
| 4 | 6,000 | ,00 | . | . | 1 | 108 |
| 5 | 7,000 | ,00 | . | . | 1 | 107 |
| 6 | 8,000 | ,00 | . | . | 1 | 106 |
| 7 | 8,000 | ,00 | . | . | 1 | 105 |
| 8 | 9,000 | ,00 | . | . | 1 | 104 |
| 9 | 12,000 | ,00 | . | . | 1 | 103 |
| 10 | 13,000 | ,00 | . | . | 1 | 102 |
| 11 | 16,000 | ,00 | . | . | 1 | 101 |
| 12 | 17,000 | 1,00 | ,981 | ,013 | 2 | 100 |
| 13 | 22,000 | 1,00 | ,971 | ,016 | 3 | 99 |
| 14 | 26,000 | ,00 | . | . | 3 | 98 |
| 15 | 30,000 | ,00 | . | . | 3 | 97 |
| 16 | 32,000 | 1,00 | . | . | 4 | 96 |
| 17 | 32,000 | 1,00 | ,951 | ,021 | 5 | 95 |
| 18 | 32,000 | ,00 | . | . | 5 | 94 |
| 19 | 33,000 | 1,00 | ,941 | ,023 | 6 | 93 |
| 20 | 35,000 | 1,00 | ,931 | ,025 | 7 | 92 |
| 21 | 36,000 | ,00 | . | . | 7 | 91 |
| 22 | 39,000 | 1,00 | . | . | 8 | 90 |
| 23 | 39,000 | 1,00 | ,911 | ,028 | 9 | 89 |
| 24 | 42,000 | 1,00 | ,900 | ,030 | 10 | 88 |
| 25 | 43,000 | 1,00 | ,890 | ,031 | 11 | 87 |
| 26 | 44,000 | ,00 | . | . | 11 | 86 |
| 27 | 44,000 | ,00 | . | . | 11 | 85 |
| 28 | 46,000 | ,00 | . | . | 11 | 84 |
| 29 | 48,000 | 1,00 | ,880 | ,033 | 12 | 83 |
| 30 | 48,000 | ,00 | . | . | 12 | 82 |
| 31 | 51,000 | 1,00 | ,869 | ,034 | 13 | 81 |
| 32 | 52,000 | 1,00 | . | . | 14 | 80 |
| 33 | 52,000 | 1,00 | ,847 | ,036 | 15 | 79 |
| 34 | 52,000 | ,00 | . | . | 15 | 78 |
| 35 | 55,000 | 1,00 | ,837 | ,037 | 16 | 77 |
| 36 | 56,000 | 1,00 | ,826 | ,039 | 17 | 76 |
| 37 | 56,000 | ,00 | . | . | 17 | 75 |
| 38 | 56,000 | ,00 | . | . | 17 | 74 |
| 39 | 57,000 | 1,00 | ,815 | ,040 | 18 | 73 |
| 40 | 59,000 | 1,00 | ,803 | ,041 | 19 | 72 |
| 41 | 61,000 | 1,00 | ,792 | ,042 | 20 | 71 |
| 42 | 64,000 | ,00 | . | . | 20 | 70 |
| 43 | 68,000 | ,00 | . | . | 20 | 69 |
| 44 | 72,000 | ,00 | . | . | 20 | 68 |
| 45 | 76,000 | ,00 | . | . | 20 | 67 |
| 46 | 80,000 | 1,00 | . | . | 21 | 66 |
| 47 | 80,000 | 1,00 | . | . | 22 | 65 |
| 48 | 80,000 | 1,00 | . | . | 23 | 64 |
| 49 | 80,000 | 1,00 | ,745 | ,045 | 24 | 63 |
| 50 | 80,000 | ,00 | . | . | 24 | 62 |
| 51 | 83,000 | 1,00 | ,733 | ,046 | 25 | 61 |
| 52 | 89,000 | 1,00 | . | . | 26 | 60 |
| 53 | 89,000 | 1,00 | ,709 | ,048 | 27 | 59 |
| 54 | 90,000 | 1,00 | ,697 | ,048 | 28 | 58 |
| 55 | 91,000 | 1,00 | ,685 | ,049 | 29 | 57 |
| 56 | 92,000 | ,00 | . | . | 29 | 56 |
| 57 | 92,000 | ,00 | . | . | 29 | 55 |
| 58 | 93,000 | 1,00 | ,672 | ,050 | 30 | 54 |
| 59 | 96,000 | ,00 | . | . | 30 | 53 |
| 60 | 99,000 | 1,00 | . | . | 31 | 52 |
| 61 | 99,000 | 1,00 | ,647 | ,051 | 32 | 51 |
| 62 | 102,000 | 1,00 | ,634 | ,051 | 33 | 50 |
| 63 | 105,000 | 1,00 | ,622 | ,052 | 34 | 49 |
| 64 | 108,000 | 1,00 | ,609 | ,052 | 35 | 48 |
| 65 | 109,000 | 1,00 | ,596 | ,053 | 36 | 47 |
| 66 | 110,000 | 1,00 | ,584 | ,053 | 37 | 46 |
| 67 | 117,000 | 1,00 | ,571 | ,054 | 38 | 45 |
| 68 | 119,000 | 1,00 | ,558 | ,054 | 39 | 44 |
| 69 | 122,000 | ,00 | . | . | 39 | 43 |
| 70 | 134,000 | ,00 | . | . | 39 | 42 |
| 71 | 137,000 | 1,00 | ,545 | ,054 | 40 | 41 |
| 72 | 139,000 | 1,00 | ,532 | ,054 | 41 | 40 |
| 73 | 140,000 | 1,00 | ,518 | ,055 | 42 | 39 |
| 74 | 145,000 | 1,00 | ,505 | ,055 | 43 | 38 |
| 75 | 148,000 | 1,00 | ,492 | ,055 | 44 | 37 |
| 76 | 152,000 | ,00 | . | . | 44 | 36 |
| 77 | 156,000 | 1,00 | ,478 | ,055 | 45 | 35 |
| 78 | 159,000 | 1,00 | ,464 | ,055 | 46 | 34 |
| 79 | 166,000 | 1,00 | ,451 | ,055 | 47 | 33 |
| 80 | 168,000 | 1,00 | ,437 | ,055 | 48 | 32 |
| 81 | 169,000 | 1,00 | . | . | 49 | 31 |
| 82 | 169,000 | 1,00 | ,410 | ,055 | 50 | 30 |
| 83 | 170,000 | 1,00 | ,396 | ,055 | 51 | 29 |
| 84 | 179,000 | 1,00 | ,382 | ,055 | 52 | 28 |
| 85 | 180,000 | 1,00 | . | . | 53 | 27 |
| 86 | 180,000 | 1,00 | ,355 | ,054 | 54 | 26 |
| 87 | 185,000 | 1,00 | ,342 | ,054 | 55 | 25 |
| 88 | 187,000 | 1,00 | ,328 | ,053 | 56 | 24 |
| 89 | 196,000 | 1,00 | . | . | 57 | 23 |
| 90 | 196,000 | 1,00 | ,301 | ,052 | 58 | 22 |
| 91 | 197,000 | 1,00 | . | . | 59 | 21 |
| 92 | 197,000 | 1,00 | ,273 | ,051 | 60 | 20 |
| 93 | 216,000 | ,00 | . | . | 60 | 19 |
| 94 | 224,000 | 1,00 | ,259 | ,050 | 61 | 18 |
| 95 | 238,000 | 1,00 | ,244 | ,049 | 62 | 17 |
| 96 | 239,000 | 1,00 | . | . | 63 | 16 |
| 97 | 239,000 | 1,00 | ,216 | ,048 | 64 | 15 |
| 98 | 243,000 | 1,00 | ,201 | ,047 | 65 | 14 |
| 99 | 247,000 | 1,00 | ,187 | ,045 | 66 | 13 |
| 100 | 251,000 | 1,00 | ,173 | ,044 | 67 | 12 |
| 101 | 260,000 | ,00 | . | . | 67 | 11 |
| 102 | 280,000 | 1,00 | ,157 | ,043 | 68 | 10 |
| 103 | 287,000 | 1,00 | ,141 | ,041 | 69 | 9 |
| 104 | 295,000 | 1,00 | ,125 | ,040 | 70 | 8 |
| 105 | 334,000 | 1,00 | ,110 | ,038 | 71 | 7 |
| 106 | 336,000 | 1,00 | ,094 | ,035 | 72 | 6 |
| 107 | 343,000 | 1,00 | ,078 | ,033 | 73 | 5 |
| 108 | 344,000 | ,00 | . | . | 73 | 4 |
| 109 | 378,000 | 1,00 | ,059 | ,030 | 74 | 3 |
| 110 | 383,000 | 1,00 | ,039 | ,026 | 75 | 2 |
| 111 | 408,000 | 1,00 | ,020 | ,019 | 76 | 1 |
| 112 | 438,000 | 1,00 | ,000 | ,000 | 77 | 0 |

| **Means and Medians for Survival Time** | | | | | |
| --- | --- | --- | --- | --- | --- |
| Mean^a^ | | | | Median | |
| Estimate | Std. Error | 95% Confidence Interval | | Estimate | Std. Error |
|  |  | Lower Bound | Upper Bound |  |  |
| 164,792 | 11,981 | 141,309 | 188,275 | 148,000 | 22,270 |

| **Means and Medians for Survival Time** | |
| --- | --- |
| Median^a^ | |
| 95% Confidence Interval | |
| Lower Bound | Upper Bound |
| 104,351 | 191,649 |

| a. Estimation is limited to the largest survival time if it is censored. |
| --- |

| **ADATRkod * ADAADApoz * CDUC Crosstabulation** | | | | | |
| --- | --- | --- | --- | --- | --- |
| CDUC | | | | ADAADApoz | Total |
|  |  |  |  | 2,00 |  |
| 1,00 | ADATRkod | ,00 | Count | 3 | 28 |
|  |  |  | % within ADATRkod | 10,7% | 100,0% |
|  |  |  | % within ADAADApoz | 100,0% | 24,6% |
|  |  | 1,00 | Count | 0 | 86 |
|  |  |  | % within ADATRkod | 0,0% | 100,0% |
|  |  |  | % within ADAADApoz | 0,0% | 75,4% |
|  | Total | | Count | 3 | 114 |
|  |  |  | % within ADATRkod | 2,6% | 100,0% |
|  |  |  | % within ADAADApoz | 100,0% | 100,0% |
| 2,00 | ADATRkod | ,00 | Count | 2 | 19 |
|  |  |  | % within ADATRkod | 10,5% | 100,0% |
|  |  |  | % within ADAADApoz | 100,0% | 44,2% |
|  |  | 1,00 | Count | 0 | 24 |
|  |  |  | % within ADATRkod | 0,0% | 100,0% |
|  |  |  | % within ADAADApoz | 0,0% | 55,8% |
|  | Total | | Count | 2 | 43 |
|  |  |  | % within ADATRkod | 4,7% | 100,0% |
|  |  |  | % within ADAADApoz | 100,0% | 100,0% |
| Total | ADATRkod | ,00 | Count | 5 | 47 |
|  |  |  | % within ADATRkod | 10,6% | 100,0% |
|  |  |  | % within ADAADApoz | 100,0% | 29,9% |
|  |  | 1,00 | Count | 0 | 110 |
|  |  |  | % within ADATRkod | 0,0% | 100,0% |
|  |  |  | % within ADAADApoz | 0,0% | 70,1% |
|  | Total | | Count | 5 | 157 |
|  |  |  | % within ADATRkod | 3,2% | 100,0% |
|  |  |  | % within ADAADApoz | 100,0% | 100,0% |
|  |  | |  |  |  |
|  |  | |  |  |  |

| **Crosstab** | | | | | |
| --- | --- | --- | --- | --- | --- |
|  | | | TLminus | | Total |
|  |  |  | ,00 | 1,00 |  |
| ADApozzz | ,00 | Count | 24 | 65 | 89 |
|  |  | % within ADApozzz | 27,0% | 73,0% | 100,0% |
|  |  | % within TLminus | 68,6% | 84,4% | 79,5% |
|  | 1,00 | Count | 11 | 12 | 23 |
|  |  | % within ADApozzz | 47,8% | 52,2% | 100,0% |
|  |  | % within TLminus | 31,4% | 15,6% | 20,5% |
| Total | | Count | 35 | 77 | 112 |
|  |  | % within ADApozzz | 31,3% | 68,8% | 100,0% |
|  |  | % within TLminus | 100,0% | 100,0% | 100,0% |

| **Crosstab** | | | | | |
| --- | --- | --- | --- | --- | --- |
|  | | | TLminus | | Total |
|  |  |  | ,00 | 1,00 |  |
| ADApoz12 | ,00 | Count | 25 | 66 | 91 |
|  |  | % within ADApoz12 | 27,5% | 72,5% | 100,0% |
|  |  | % within TLminus | 71,4% | 85,7% | 81,3% |
|  | 1,00 | Count | 4 | 10 | 14 |
|  |  | % within ADApoz12 | 28,6% | 71,4% | 100,0% |
|  |  | % within TLminus | 11,4% | 13,0% | 12,5% |
|  | 2,00 | Count | 6 | 1 | 7 |
|  |  | % within ADApoz12 | 85,7% | 14,3% | 100,0% |
|  |  | % within TLminus | 17,1% | 1,3% | 6,3% |
| Total | | Count | 35 | 77 | 112 |
|  |  | % within ADApoz12 | 31,3% | 68,8% | 100,0% |
|  |  | % within TLminus | 100,0% | 100,0% | 100,0% |

| **Crosstab** | | | | | |
| --- | --- | --- | --- | --- | --- |
|  | | | LORkummulativ | | Total |
|  |  |  | ,00 | 1,00 |  |
| ADApozzz | ,00 | Count | 71 | 18 | 89 |
|  |  | % within ADApozzz | 79,8% | 20,2% | 100,0% |
|  |  | % within LORkummulativ | 85,5% | 62,1% | 79,5% |
|  | 1,00 | Count | 12 | 11 | 23 |
|  |  | % within ADApozzz | 52,2% | 47,8% | 100,0% |
|  |  | % within LORkummulativ | 14,5% | 37,9% | 20,5% |
| Total | | Count | 83 | 29 | 112 |
|  |  | % within ADApozzz | 74,1% | 25,9% | 100,0% |
|  |  | % within LORkummulativ | 100,0% | 100,0% | 100,0% |

| **Chi-Square Tests** | | | |
| --- | --- | --- | --- |
|  | Value | df | Asymp. Sig. (2-sided) |
| Pearson Chi-Square | 7,256^a^ | 1 | ,007 |
| Continuity Correction^b^ | 5,889 | 1 | ,015 |
| Likelihood Ratio | 6,648 | 1 | ,010 |
| Fisher's Exact Test |  |  |  |
| Linear-by-Linear Association | 7,192 | 1 | ,007 |
| N of Valid Cases | 112 |  |  |

**tranziens * LORkummulativ**

| **Crosstab** | | | | | |
| --- | --- | --- | --- | --- | --- |
|  | | | LORkummulativ | | Total |
|  |  |  | ,00 | 1,00 |  |
| tranziens | ,00 | Count | 80 | 25 | 105 |
|  |  | % within tranziens | 76,2% | 23,8% | 100,0% |
|  |  | % within LORkummulativ | 96,4% | 86,2% | 93,8% |
|  | 1,00 | Count | 3 | 4 | 7 |
|  |  | % within tranziens | 42,9% | 57,1% | 100,0% |
|  |  | % within LORkummulativ | 3,6% | 13,8% | 6,3% |
| Total | | Count | 83 | 29 | 112 |
|  |  | % within tranziens | 74,1% | 25,9% | 100,0% |
|  |  | % within LORkummulativ | 100,0% | 100,0% | 100,0% |

| **Chi-Square Tests** | | | |
| --- | --- | --- | --- |
|  | Value | df | Asymp. Sig. (2-sided) |
| Pearson Chi-Square | 3,800^a^ | 1 | ,051 |
| Continuity Correction^b^ | 2,261 | 1 | ,133 |
| Likelihood Ratio | 3,289 | 1 | ,070 |
| Fisher's Exact Test |  |  |  |
| Linear-by-Linear Association | 3,766 | 1 | ,052 |
| N of Valid Cases | 112 |  |  |

| **Chi-Square Tests** | | |
| --- | --- | --- |
|  | Exact Sig. (2-sided) | Exact Sig. (1-sided) |
| Pearson Chi-Square |  |  |
| Continuity Correction^b^ |  |  |
| Likelihood Ratio |  |  |
| Fisher's Exact Test | ,073 | ,073 |
| Linear-by-Linear Association |  |  |
| N of Valid Cases |  |  |

| a. 1 cells (25,0%) have expected count less than 5. The minimum expected count is 1,81. |
| --- |
| b. Computed only for a 2x2 table |

| **Risk Estimate** | | | |
| --- | --- | --- | --- |
|  | Value | 95% Confidence Interval | |
|  |  | Lower | Upper |
| Odds Ratio for tranziens (,00 / 1,00) | 4,267 | ,894 | 20,362 |

**dozismodif * LORkummulativ**

| **Crosstab** | | | | | |
| --- | --- | --- | --- | --- | --- |
|  | | | LORkummulativ | | Total |
|  |  |  | ,00 | 1,00 |  |
| dozismodif | ,00 | Count | 65 | 14 | 79 |
|  |  | % within dozismodif | 82,3% | 17,7% | 100,0% |
|  |  | % within LORkummulativ | 78,3% | 48,3% | 70,5% |
|  | 1,00 | Count | 18 | 15 | 33 |
|  |  | % within dozismodif | 54,5% | 45,5% | 100,0% |
|  |  | % within LORkummulativ | 21,7% | 51,7% | 29,5% |
| Total | | Count | 83 | 29 | 112 |
|  |  | % within dozismodif | 74,1% | 25,9% | 100,0% |
|  |  | % within LORkummulativ | 100,0% | 100,0% | 100,0% |

| **Chi-Square Tests** | | | |
| --- | --- | --- | --- |
|  | Value | df | Asymp. Sig. (2-sided) |
| Pearson Chi-Square | 9,330^a^ | 1 | ,002 |
| Continuity Correction^b^ | 7,941 | 1 | ,005 |
| Likelihood Ratio | 8,830 | 1 | ,003 |
| Fisher's Exact Test |  |  |  |
| Linear-by-Linear Association | 9,247 | 1 | ,002 |
| N of Valid Cases | 112 |  |  |

| **Chi-Square Tests** | | |
| --- | --- | --- |
|  | Exact Sig. (2-sided) | Exact Sig. (1-sided) |
| Pearson Chi-Square |  |  |
| Continuity Correction^b^ |  |  |
| Likelihood Ratio |  |  |
| Fisher's Exact Test | ,004 | ,003 |
| Linear-by-Linear Association |  |  |
| N of Valid Cases |  |  |

| a. 0 cells (,0%) have expected count less than 5. The minimum expected count is 8,54. |
| --- |
| b. Computed only for a 2x2 table |

| **Risk Estimate** | | | |
| --- | --- | --- | --- |
|  | Value | 95% Confidence Interval | |
|  |  | Lower | Upper |
| Odds Ratio for dozismodif (,00 / 1,00) | 3,869 | 1,579 | 9,479 |
| For cohort LORkummulativ = ,00 | 1,508 | 1,087 | 2,094 |
| For cohort LORkummulativ = 1,00 | ,390 | ,213 | ,714 |
| N of Valid Cases | 112 |  |  |

| **Crosstab** | | | | | |
| --- | --- | --- | --- | --- | --- |
|  | | | nem | | Total |
|  |  |  | 1,00 | 2,00 |  |
| LORkummulativ | ,00 | Count | 46 | 37 | 83 |
|  |  | % within LORkummulativ | 55,4% | 44,6% | 100,0% |
|  |  | % within nem | 92,0% | 59,7% | 74,1% |
|  | 1,00 | Count | 4 | 25 | 29 |
|  |  | % within LORkummulativ | 13,8% | 86,2% | 100,0% |
|  |  | % within nem | 8,0% | 40,3% | 25,9% |
| Total | | Count | 50 | 62 | 112 |
|  |  | % within LORkummulativ | 44,6% | 55,4% | 100,0% |
|  |  | % within nem | 100,0% | 100,0% | 100,0% |

| **Chi-Square Tests** | | | |
| --- | --- | --- | --- |
|  | Value | df | Asymp. Sig. (2-sided) |
| Pearson Chi-Square | 15,070^a^ | 1 | ,000 |
| Continuity Correction^b^ | 13,433 | 1 | ,000 |
| Likelihood Ratio | 16,623 | 1 | ,000 |
| Fisher's Exact Test |  |  |  |
| Linear-by-Linear Association | 14,936 | 1 | ,000 |
| N of Valid Cases | 112 |  |  |

| **Chi-Square Tests** | | |
| --- | --- | --- |
|  | Exact Sig. (2-sided) | Exact Sig. (1-sided) |
| Pearson Chi-Square |  |  |
| Continuity Correction^b^ |  |  |
| Likelihood Ratio |  |  |
| Fisher's Exact Test | ,000 | ,000 |
| Linear-by-Linear Association |  |  |
| N of Valid Cases |  |  |

| a. 0 cells (,0%) have expected count less than 5. The minimum expected count is 12,95. |
| --- |
| b. Computed only for a 2x2 table |

| **Risk Estimate** | | | |
| --- | --- | --- | --- |
|  | Value | 95% Confidence Interval | |
|  |  | Lower | Upper |
| Odds Ratio for LORkummulativ (,00 / 1,00) | 7,770 | 2,483 | 24,315 |
| For cohort nem = 1,00 | 4,018 | 1,585 | 10,185 |
| For cohort nem = 2,00 | ,517 | ,391 | ,685 |
| N of Valid Cases | 112 |  |  |
